# Travel patterns during pregnancy: comparison between Global Positioning System (GPS) tracking and questionnaire data

**DOI:** 10.1186/1476-069X-12-86

**Published:** 2013-10-09

**Authors:** Jun Wu, Chengsheng Jiang, Guillermo Jaimes, Scott Bartell, Andy Dang, Dean Baker, Ralph J Delfino

**Affiliations:** 1 Program in Public Health, College of Health Sciences, University of California, Irvine, CA, USA; 2 Department of Epidemiology, School of Medicine, University of California, Irvine, CA, USA; 3 Maryland Institute for Applied Environmental Health, School of Public Health, University of Maryland, College Park, MD, USA; 4 Department of Environmental Science, Policy, & Management, University of California, Berkeley, CA, USA; 5 Center for Occupational & Environmental Health, University of California, Irvine, CA, USA

## Abstract

**Background:**

Maternal exposures to traffic-related air pollution have been associated with adverse pregnancy outcomes. Exposures to traffic-related air pollutants are strongly influenced by time spent near traffic. However, little is known about women’s travel activities during pregnancy and whether questionnaire-based data can provide reliable information on travel patterns during pregnancy.

**Objectives:**

Examine women’s in-vehicle travel behavior during pregnancy and examine the difference in travel data collected by questionnaire and global positioning system (GPS) and their potential for exposure error.

**Methods:**

We measured work-related travel patterns in 56 pregnant women using a questionnaire and one-week GPS tracking three times during pregnancy (<20 weeks, 20–30 weeks, and >30 weeks of gestation). We compared self-reported activities with GPS-derived trip distance and duration, and examined potentially influential factors that may contribute to differences. We also described in-vehicle travel behavior by pregnancy periods and influences of demographic and personal factors on daily travel times. Finally, we estimated personal exposure to particle-bound polycyclic aromatic hydrocarbon (PB-PAH) and examined the magnitude of exposure misclassification using self-reported vs. GPS travel data.

**Results:**

Subjects overestimated both trip duration and trip distance compared to the GPS data. We observed moderately high correlations between self-reported and GPS-recorded travel distance (home to work trips: r = 0.88; work to home trips: r = 0.80). Better agreement was observed between the GPS and the self-reported travel time for home to work trips (r = 0.77) than work to home trips (r = 0.64). The subjects on average spent 69 and 93 minutes traveling in vehicles daily based on the GPS and self-reported data, respectively. Longer daily travel time was observed among participants in early pregnancy, and during certain pregnancy periods in women with higher education attainment, higher income, and no children. When comparing self-reported vs. GPS data, we found that estimated personal exposure to PB-PAH did not differ remarkably at the population level, but the difference was large at an individual level.

**Conclusion:**

Self-reported home-to-work data overestimated both trip duration and trip distance compared to GPS data. Significant differences in PAH exposure estimates were observed at individual level using self-reported vs. GPS data, which has important implications in air pollution epidemiological studies.

## Background

There is a growing concern about the health impact of traffic-related air pollution on pregnancy outcomes [1-4]. Living close to freeways or high-traffic density areas have been associated with spontaneous abortion, pregnancy hypertension, preterm birth, and term low birth weight [5-8]. Several recent studies further reported that maternal exposure to traffic-related air pollutants is associated with risk of preeclampsia, reduced fetal growth, preterm birth, small for gestational age, and term low birth weight [2,9-11].

Exposures to traffic-related pollutants are strongly influenced by time spent near traffic emission sources (e.g. in-vehicle travel and walking). Concentrations of ultrafine particles and volatile organic compounds can be up to ten times higher in vehicles than in ambient outdoor environments [12-16]. It has been estimated that around 33-45% of ultrafine particles and 30-55% of black carbon exposure for nonsmoking urbanites in Los Angeles comes from time in vehicles [15,17]. We conducted a personal exposure measurement study and reported that in-vehicle travel time explained approximately 40% of the variance in daily personal exposure to particle-bound polycyclic aromatic hydrocarbon [18]. However, only two studies in Southern California specifically examined exposure to traffic-related air pollutants from time in transit in relation to health outcomes [19,20]. Ritz and Yu (1999) found higher risk of term low birth weight for women who commuted more than 60 minutes to work using a census-based measure of commuting level (although no individual data were available and no dose–response relation was reported). McConnell et al. (2010) reported the risk of severe wheeze was associated with commuting time in asthmatic children; the association was stronger in analysis restricted to children with commuting times 5 minutes or longer.

Because of the potentially high air pollutant exposures in transit environments, it is essential to understand pregnant women’s travel behaviors for more accurate exposure assessment. However, few time-activity studies have focused on pregnant women, and little is known about travel behavior during pregnancy. The National Human Activity Patterns Survey (NHAPS) (the largest time-activity study in the U.S.) collected over 9,300 time-activity surveys but did not address pregnancy status [21]. A number of other studies have examined exercise and physical activities of pregnant women [22-25], but with no focus on time in traffic. A Canadian study examined the change in location-based activity patterns during pregnancy, but it relied on a self-reported time-activity log and focused on time spent at home rather than in transit [26].

Conventional methods for time-location collections (e.g. self-reported paper diary and telephone interview) have several major limitations, including omission of short trips and inaccurate reporting of trip duration [27,28]. Global positioning system (GPS) techniques have been increasingly used to track people’s time-location or commuting patterns [29-34]. GPS tracking has the advantages of continuous recording, high temporal resolution, and minimum reporting burden for participants [35]. However, sometimes GPS tracking is not an option in many epidemiological studies due to concerns about the protection of confidentiality in human subjects, cost considerations, or the study design (e.g. retrospective studies and interest in long-term exposures). Under such conditions, epidemiological studies have to rely on questionnaires to obtain time-activity data. Little information is available in the literature on how questionnaire and GPS tracking compare with each other in data quality. In addition, few epidemiological studies have effectively used GPS data for time-activity pattern classification, likely due to issues including the quality of GPS data, the compliance of human subjects, and the lack of reliable methods to mine raw GPS data [36].

The objectives of this paper are to examine the in-vehicle work-related travel behavior of pregnant women at different stages of pregnancy (<20 weeks, 20–30 weeks, and >30 weeks of gestation), examine the difference in travel time collected by two instruments (i.e. questionnaire and GPS tracking), investigate influential factors contributing to the difference of travel time, and examine potential exposure error in estimating personal particle-bound polycyclic aromatic hydrocarbon (PB-PAH) using the two instruments collecting travel data. PAH has been linked to adverse health effects, including adverse birth outcomes [37] and allergy and asthma in children [38].

## Method

### Population

We recruited 92 pregnant women before 20 weeks of gestation at two hospitals (Long Beach Memorial Medical Center and Medical Center of University of California, Irvine) in South Los Angeles County and Orange County, California in 2009–2010. Women were recruited mainly through brochures and flyers at the hospitals and a few subjects (N = 5) through word of mouth from other subjects. Eligibility criteria included age 18 years or older, nonsmoker, and low-risk pregnancy (e.g. excluding those with illegal drug use, alcohol abuse, hypertension or diabetes before pregnancy). For the present data analysis of work-related commuting and exposure, we included 56 subjects who worked during pregnancy. Gestational age was calculated based on a combination of self-reported and doctor diagnosed date. Twenty-eight out of 92 subjects participated in a personal PB-PAH exposure assessment study [18], which provides the basis for PB-PAH exposure modeling in this paper. The study protocol and material was approved by the University of California, Irvine Institutional Review Board for biomedical research.

### Questionnaire interview

After consenting subjects, our research staff visited the home of each subject and administered a baseline questionnaire on demographic and socioeconomic (SES) information and an environmental and behavior questionnaire on travel patterns and other risk factors during pregnancy. The baseline questionnaire documented age, reproductive history, education, annual family income, marital status, primary language spoken at home, and race/ethnicity of the subject. The environmental and behavior questions were administered three times during pregnancy (before 20 weeks, 20–30 weeks, and >30 weeks of gestation) and asked the typical environmental and behavior patterns of the pregnant women in the past three months of the interview day. The information we collected included home location (both address and GPS coordinates) and work locations (address), regular work days, transportation mode from home to work, trip duration and distance for home to work commute and vice versa, and average daily in-vehicle travel time for non-work related trips. The questions we used in this study are listed in Additional file 1.

### GPS tracking

After each environmental and behavior interview, each study participant was asked to carry a portable GlobalSat DG-100 GPS device (approximately 227 g and was placed in her purse or a shoulder-style messenger bag) during waking hours for seven consecutive days (1 week) starting the next day after the interview. This GPS device has been used in another human time-activity study in Southern California [39] and has been shown to have good spatial accuracy and reliable performance [40]. Since the battery life of the GPS device is approximately 17 hours, the participants were asked to turn on the device when they woke up and turn it off at the end of their day to charge the battery. GPS recordings are often incomplete because of subject noncompliance, short battery life (e.g. the subjects forgot to shut down and recharge the device at night), mechanical failure, and the block of satellite signals by buildings and other structures. Therefore, we identified days with sufficiently complete GPS data for the present analysis. In this study, we defined a valid GPS day as a 16-hr day (7:00 AM to 10:59 PM) with no more than 50% of expected GPS data that was missing, equivalent to ≥8 hours of GPS data during typical waking hours. The valid GPS data was used to both maintain data quality and maximize data retention for analysis. Previous studies of GPS activity tracking have used various criteria for a valid GPS day. Troped et al. [41] applied a cut-off of 1 standard deviation below the mean of recorded daily data (40 minutes). Cooper [42] included data with ≥3 h of outdoor GPS and accelerometer recordings per day for ≥1 day. Almanza [43] included data with ≥4 h of GPS and accelerometer recordings per day for ≥3 days. We used a more stringent criterion than the previous studies since we focused on in-vehicle travel which may occur infrequently while the other studies focused on physical activities of the study subjects. In addition, we aimed to examine not only trip level data but also daily average travel time, and a longer time average would be more appropriate for the latter.

### Vehicle trip classification

We classified the GPS points into four major time-activity categories: indoor, outdoor static, outdoor walking and in-vehicle travel using a rule-based automated method described by us elsewhere [36]. With high-quality training and validation data, we reported that the model had 87.8% sensitivity, 99.5% specificity, and 89.1% precision in identifying in-vehicle travel GPS points [36]. Trips were extracted for continuous in-vehicle travel points from the model output. Locations of subjects’ home and workplaces were obtained from address data using the TeleAtlas Geocoding Service and from GPS recordings (home location only). The following procedures were performed to classify trips based on in-vehicle travel points identified from our automation model:

1) Adjacent trips were consolidated if the end of the earlier trip and the start of the latter trip were within 2 minutes in time and 250 m in distance.

2) The start and end point of each trip was assigned to a home or a work location if it was within 350 m of a GPS-based home location or 500 m of a geocoded workplace location identified from the subject questionnaire, respectively; otherwise, it was assigned as other locations. We assumed that the GPS records had better quality than the geocoded addresses.

3) Since subjects may stop to drop or pick up their children or run short errands on the way from home to work or back, we further consolidated adjacent trips in time (i.e., the gap did not contribute to the duration of the one trip) if they satisfied the following criteria:

• The trip is not directly home to work or work to home;

• Time gap between the two trips is small (i.e. less than 15 minutes for home-originated trip and 30 minutes for work-originated trip) since longer gap time likely indicates a different trip.

4) After the above procedures, we further excluded the trips that lasted for no more than two minutes since such short trips are likely trips misclassified by our time-activity model [36].

Next, we extracted all the GPS points of each trip and overlayed them with the 2003 TeleAtlas® street data using ArcGIS 9.3 (ESRI, Redlands, CA). We then calculated the shortest distance between each GPS point to the freeway network. A GPS point was assumed to be on freeway if it was within 50 m of a freeway. Finally, we calculated the percentage of travel time on freeways for each individual trip and each sampling day and week.

### Data analysis

Statistical analyses were conducted in SAS 9.2 (SAS Institute Inc., Cary, NC) and R 2.10.1 (R Development Core Team, Vienna, Austria). Differences between the GPS and questionnaire data were examined for distance and duration of work-related trips and for average daily travel time. We did not thoroughly examine non-work related travels in working subjects because it was not the focus of the study (only two questions addressed non-work related trips). In addition, our trip classification algorithm may misclassify certain work-related trips (e.g. if incompletely recorded by the GPS device) as non-work trips, leading to the overestimation of travel time for non-work related trips. ANOVA analysis was further conducted to compare GPS-derived total daily travel time among different pregnancy periods and by different demographic and socioeconomic variables (e.g. age, household income, working status, marital status, and parity) among different pregnancy periods.

We developed linear regression models and linear mixed effect models to examine the difference between self-reported and GPS-based trip duration for home to work and work to home trips. The variables we examined in the models included questionnaire-based travel information (e.g. self-reported trip distance and duration and the GPS-based percentage of travel time on freeways), socio-demographic variables (age, working status, education, income, marital status, number of children and total number of persons in a household, and parity), and other factors that may influence travel time (season, day of week, rush hour, and the percent of travel time on freeways based on GPS data). The following parameters were treated as binary variables: 6-month season (cool or warm), day of week (weekday or weekend), trip starting in rush hours (yes or no), age (<30 or ≥30), household income (<$50 k or ≥ $50 k), marital status (yes or no), number of children (0 or ≥1) and the total number of persons (≤2 or >2) in a household, and parity (0 or ≥1). We classified May to October as warm season and November to April as cool season. The rush hour variable was assigned to trips starting between 6 AM and 8 AM or between 4 PM and 7 PM on weekdays (32% of the home to work trips and 50% of work to home trips). A sensitivity analysis was conducted to examine various definitions of rush hour.

We first examined the correlation of each predictor variable with the outcome variable (individual trip duration or daily travel time). A predictor variable was dropped from further analysis if the absolute correlation coefficient with the outcome variable was less than 0.10. We used the LASSO method of variable selection in the SAS GLMSELECT procedure to select the best-fit linear model. The models were further checked by the variance inflation factor statistics (VIF) to assess potential colinearity. Leave-one-out cross-validation (CV) was used to evaluate the models. This method used one sample for validation and used the remaining data for training and this process iterated for all the samples. We calculated R^2^ of all the estimated versus observed values and the square root of the mean of the squared errors (RMSE). A key advantage of the linear model is an easily interpreted R^2^, but it does not account for within-subject correlations. Therefore, we also fit linear mixed effect models using the same set of variables with a random intercept and random slope for each subject. We compared the performance of the linear and the mixed effect models using likelihood ratio tests in R (anova function).

Finally, we examined potential exposure misclassification caused by the difference in self-reported and GPS-derived travel time data by estimating personal exposures to PB-PAH in the study participants based on a PB-PAH personal exposure model developed from our previous work [18]. We selected PB-PAH because it is an important air pollutant from direct traffic emissions and it has been linked to adverse health effects [37,38]. Further, regression models have been developed by us previously to estimate personal PAH exposures based on GPS data in pregnant women or women who had delivered babies within one year of the sampling dates [18]. Briefly, we sampled real-time personal PB-PAH exposure coupling with GPS time-activity tracking for a subset of 28 subjects who came from the same cohort as the subjects in this study. Measurements were conducted one to three times and one to nine days each time from August 2009 to November 2010. In addition, each subject filled out a baseline questionnaire on demographic and socioeconomic information and an additional questionnaire on major environmental and behavior patterns that may influence personal PB-PAH exposure in the past three months of the sampling. Regression models (adjusted R^2^ ranging from 0.58 to 0.75) were developed to estimate weekly, daily, and microenvironmental PB-PAH exposures based on GPS-tracking data, traffic activity, roadway data, and simple questionnaire information. Since we focused on trimester-average exposure in this study, we selected the model with the longest averaging time (weekly). The model had an adjusted R^2^ of 0.61 and used three variables (percent of in-vehicle travel time, percent of weekday time, had work-related exposure to traffic pollutants) [18]. More details about the model can be found elsewhere [18]. Since we aimed to understand the reliability of travel time on personal exposure estimates, we kept the other two variables constant by assuming that there was no occupational exposure among the subjects and that each subject worked five days per week. Personal weekly PB-PAH exposure was then estimated for each subject based first on the percent of in-vehicle travel time derived from the GPS data (i.e. dividing the total GPS-identified in-vehicle travel time by the total GPS time in a sampling week) and then on the self-reported data (i.e. dividing the sum of self-reported home to work, work to home, and non-work related daily travel time by 24 hours). Sensitivity analysis was also conducted to examine the magnitude of exposure misclassification if only work-related travel was considered in exposure assessment.

## Results

Subjects had a diverse SES background, with approximately half of the women having an educational attainment of up to high school and about 35% of women had a household income below $30,000 (Table 1). Of the 56 subjects, 7, 12, and 32 subjects had missing or invalid GPS data before 20 weeks, 20–30 weeks, and more than 30 weeks of gestation, respectively (Table 2). Thirty-three subjects had ≥3 valid GPS days of data for at least two pregnancy periods. As expected, the percent of women who worked decreased significantly with the advance of pregnancy (49 subjects before 20 weeks and 24 subjects after 30 weeks of gestation). Among different pregnancy periods we observed no remarkable change in the availability of a car for transportation, and the percentage of women reported going to work by walking (11.4%-12.5%) or by bus, metro rail, or train (6.8% - 14.3%).

**Table 1:** Statistics of the study subjects at baseline (<20 weeks of gestation) who had questionnaire and at least one complete day of GPS tracking data (N = 56)

**Variable**	**Level**	**N (%)**
Age	<20	3 (5.4)
	20-29	25 (44.6)
	30-39	27 (48.2)
	40	1 (1.8)
Marital status	Yes	33 (58.9)
	No	23 (41.1)
Number of other children	0	30 (53.6)
	1	16 (28.6)
	>1	10 (17.9)
Education level	Lower than high school	5 (8.9)
	High school	19 (33.9)
	Technical/Trade school	8 (14.3)
	College or university	15 (26.8)
	Master or higher degree	9 (16.1)
Annual household income	<$30,000	20 (35.7)
	$30,000-$50,000	9 (16.1)
	$50,000-$75,000	8 (14.3)
	>$75,000	18 (32.1)
	Don’t Know	1 (1.8)
Ethnicity	Asian and Pacific Islanders	7 (12.5)
	Hispanic	28 (50)
	Non-Hispanic White	16 (28.6)
	Other	5 (8.9)

**Table 2:** **Number and percentage of the subjects (self-reported workers) who had both questionnaire and GPS tracking data for at least one valid GPS day**^***a***^

	**Any pregnancy period**	**<20 weeks**	**20-30 weeks**	**>30 weeks**	**Two or three pregnancy periods**	**All three pregnancy periods**	**Total person-weeks**
Number of subjects	56 (100.0%)	49 (100.0%)	44 (100.0%)	24 (100.0%)	45 (100.0%)	16 (100.0%)	117
Had ≥2 valid GPS days	55 (98.2%)	44 (89.8%)	38 (86.4%)	21 (87.5%)	38 (84.4%)	10 (62.5%)	103
Had ≥3 valid GPS days	52 (92.9%)	40 (81.6%)	35 (79.5%)	18 (75.0%)	33 (73.3%)	8 (50.0%)	93
Had a car for transportation	54 (96.4%)	46 (93.9%)	40 (90.9%)	21 (87.5%)	40 (88.9%)	13 (81.3%)	107
Going to work by automobile	51 (91.1%)	44 (89.8%)	39 (88.6%)	22 (91.7%)	41 (91.1%)	13 (81.3%)	105
Going to work by bus, metro rail, or train	7 (12.5%)	7 (14.3%)	3 (6.8%)	3 (12.5%)	3 (6.7%)	3 (18.8%)	13
Going to work by walking	7 (12.5%)	6 (12.2%)	5 (11.4%)	3 (12.5%)	5 (11.1%)	2 (12.5%)	14

^*a*^Valid GPS day: ≥50% of GPS data between 7:00 AM and 10:59 PM.

Based on the questionnaire data, subjects who commuted by automobiles spent an average of 20.8 minutes and 18.3 km for home to work commute and 27.0 minutes and 21.2 km for work to home commute per day (assuming one home to work and one work to home trip per day) on working days (Table 3). They reported an average of 34.3 minutes and 26.9 km traveling in vehicles daily if weighted by both the number of working days plus non-working days for each subject on a 7-day basis. They also reported an additional 53 minutes daily traveling in vehicles for non-work trips (for all days).

**Table 3:** **Summary of the questionnaire and the GPS data**^***a ***^**on travel distance and duration averaged by each subject sampling week for workers who commuted by automobiles and had GPS work-related trips (N = 51 person-weeks or 124 person-days)**

	**Home to work commute**				**Work to home commute**			
	**Self-reported trip distance (km)**	**GPS-based trip distance (km)**	**Self-reported trip duration (minute)**	**GPS-based trip duration (minute)**	**Self-reported trip distance (km)**	**GPS-based trip distance (km)**	**Self-reported trip duration (minute)**	**GPS-based trip duration (minute)**
Number of person-weeks	44	45	45	45	41	44	44	44
Trip-level statistics								
Minimum	1.6	1.7	4.0	3.5	1.6	1.8	5.0	3.6
Maximum	64.4	59.6	60.0	99.9	88.5	62.9	90.0	106.6
Mean	18.3	17.3	20.8	19.9	21.2	18.7	27.0	22.8
Standard deviation	16.7	15.1	13.8	18.1	22.4	15.7	21.8	17.9
Daily mean^*b*^	12.9	NA	16.5	NA	14.0	NA	17.8	NA
	Pearson’s correlation coefficient							
Self-reported trip distance	1.00				1.00			
GPS-based trip distance	0.88	1.00			0.80	1.00		
Self-reported trip duration	0.85	0.85	1.00		0.93	0.77	1.00	
GPS-based trip duration	0.78	0.84	0.77	1.00	0.68	0.87	0.64	1.00

^*a*^Based on data from valid GPS days (≥50% of GPS data between 7:00 AM and 10:59 PM).

^*b*^Weighted average of the trip mean by the number of working and non-working days for subjects (mean = 4.3 days; range: 1–7 days).

Moderately high correlation was observed between self-reported trip distance and trip duration (r = 0.85-0.93), GPS-based trip distance and trip duration (r = 0.84-0.87), and self-reported and GPS-based trip distance (r = 0.80-0.88) (Table 3). For trip duration, we observed better agreement between self-reported and the GPS data for home to work trips than work to home trips (r = 0.77 vs. 0.64). Compared to GPS, self-reported data overestimated both trip duration and trip distance by approximately 5% for home to work trips, 13.3% for work to home trip distance, and 18.5% for work to home trip duration (Table 3). The difference was significant for home to work trip distance (p-value: 0.03) and marginally significant for work to home trip duration (p-value: 0.11), but insignificant for home to work trip duration (p-value: 0.32) and work to home distance (p-value: 0.97).

Models explained approximately 30% of the variance for the difference of in-vehicle travel time measured by self-reported vs. GPS method (Table 4). For home to work trips (N = 93 trips), self-reported trip distance and trip starting in rush hour were negatively associated with the difference between the self-reported and GPS travel time, while the use of a Japanese-made vehicle, more than two persons in the household, and percentage of travel time on freeways were positively associated with the difference. For work to home trips (N = 89 trips), self-reported trip duration was positively associated with the difference between self-reported and trip travel time, while weekday was negatively associated with the difference. The linear regression and the mixed effect model produced similar results although the likelihood ratio tests had p-values <0.001 for both models, indicating that a random effect model had a much better fit than the linear model.

**Table 4:** Linear and mixed effect models to estimate the differences in GPS-based and self-reported travel time (self-reported – GPS) for home to work and work to home trips

	**General linear regression**					**Mixed effect model (fixed effects)**		
	**Beta**	**Standard error**	**p-value**	**Partial R**^**2**^	**VIF**	**Beta**	**Standard error**	**p-value**
Home to work trips (93 trips^*a*^ from 31 subjects)	(Model R^2^ = 0.33; cross validation R^2^ = 0.29)							
Intercept	-17.62	5.11	0.0009		0	-17.16	8.13	0.0439
Self-reported distance for home to work trips	-0.27	0.08	0.0011	0.09	1.53	-0.28	0.12	0.0196
Vehicle make (Japanese car: 1; other: 0)	7.81	2.24	0.0008	0.09	1.07	7.24	3.44	0.0397
Rush hour (yes/no)	-7.42	2.52	0.0041	0.07	1.07	-5.97	2.29	0.0114
More than two persons in the household (yes/no)	6.52	2.29	0.0055	0.04	1.06	5.72	3.48	0.1054
Percentage of travel time on freeways	16.12	4.43	0.0005	0.04	1.56	19.49	5.78	0.0013
Work to home trips (89 trips from 31 subjects)	(Model R^2^ = 0.36; cross validation R^2^ = 0.31)							
Intercept	0.71	4.50	0.8752		0	0.45	4.91	0.9272
Self-reported duration for work to home trips	0.48	0.07	<.0001	0.32	1.00	0.46	0.10	<.0001
Weekday (yes/no)	-10.80	4.43	0.0165	0.04	1.00	-10.57	3.99	0.0105

^*a*^Excluding four trips with missing self-reported distance.

Based on the GPS data with at least two valid GPS days in each sampling week, the subjects on average spent 73, 66, and 64 minutes daily traveling in vehicles before 20 weeks, 20–30 weeks, and more than 30 weeks of gestation, respectively (Table 5). These subjects on average spent 69 minutes and 93 minutes daily traveling in vehicles (including non-work trips) based on the GPS and the self-reported data, respectively. GPS-based daily travel time was significantly higher in women who had university or above degrees compared to those with lower than high school or technical or trade school degree (significant for <20 weeks and marginally significant for 20–30 weeks), in women who had no children (<20 weeks), and in women who had higher income (20–30 weeks). No substantial difference was observed in daily travel time by age, marital status, race and ethnicity, or number of persons in the household. Among these subjects having data for at least two pregnancy periods, we found that on a daily basis they traveled 29 minutes longer in vehicles in early pregnancy than late pregnancy (N = 12, p-value: 0.01) and 8 minutes longer in early pregnancy than mid-pregnancy (N = 26, p-value: 0.11). The difference was less than four minutes between mid-pregnancy and late pregnancy (N = 13, p-value: 0.61) for the daily travel time.

**Table 5:** **ANOVA analysis of total daily in-vehicle travel time based on GPS data**^***a ***^**by subject characteristics**

		**<20 weeks**			**20-30 weeks**			**>30 weeks**		
**Variable**	**Values**	**N**	**Travel time (min/day)**	**p-value**	**N**	**Travel time (min/day)**	**p-value**	**N**	**Travel time (min/day)**	**p-value**
All subjects		42	73		36	66		19	64	
Age	<30	21	63	0.09	16	58	0.14	10	61	0.64
	≥30	21	84		20	73		9	68	
Education level	High school	18	69	0.00	13	54	0.06	11	62	0.58
	University and above	18	87		20	76		7	71	
	Other^*b*^	6	44		3	54		1	47	
Number of other children	0	20	87	0.03	21	72	0.28	12	72	0.21
	≥1	22	61		15	59		7	52	
Number of persons	≤2	22	83	0.08	22	71	0.36	13	70	0.34
	>2	20	63		14	60		6	53	
Annual household income	≤$50,000	22	65	0.10	18	56	0.02	11	58	0.30
	>$50,000	19	85		17	80		8	73	
	Unknown	1	38		1	15				
Marital status	Yes	25	79	0.24	22	68	0.79	11	68	0.55
	No	17	65		14	64		8	59	
Ethnicity	Asian	4	79	0.69	6	70	0.52	3	51	0.21
	Hispanic	18	68		16	62		8	66	
	White	15	82		11	66		5	61	
	Other	5	64		3	82		3	80	

^*a*^Averaged for each person-week with at least two valid GPS days (valid GPS day: ≥50% of GPS data between 7:00 AM and 10:59 PM).

^*b*^Technical school, or trade school, or lower than high school.

Among 48 subjects (N = 79 person-weeks) who had both self-reported and GPS-based in-vehicle travel time data, we estimated an average weekly personal PB-PAH exposure of 11.00 μg/m^3^ and 9.50 μg/m^3^ based on self-reported and GPS travel time, respectively. Compared to the GPS data, the self-reported data on average overestimated exposure by 15.8% for the study population and misclassified exposure by -44.0 to 308.1% (mean: 19.2%; standard deviation: 0.44) for individual subjects. However, we note that GPS is not a perfect gold standard here since some in-vehicle travel may have been missed in the GPS data although we restricted the analysis to only valid GPS days with ≥8 hours of data during the typical waking hours. Among a subset of 28 subjects (N = 37 person-weeks) who had both self-reported and GPS-based home to work and work to home travel time data, we estimated an average weekly personal PB-PAH exposure of 8.58 μg/m^3^ and 8.09 μg/m^3^ based on self-reported and GPS-based data, respectively. Compared to the GPS data, the self-reported data on average overestimated exposure by 6.1% and misclassified exposure by -21.6 to 40.3% (mean: 6.6%; standard deviation: 0.13) for individual in this subset. Finally, the exclusion of non-work trips among these 28 subjects underestimated the exposure by 28% and 14% based on questionnaire and GPS data, respectively.

## Discussion

To our knowledge, this is the first study that has examined in-vehicle travel patterns in pregnant women based on both a self-administered questionnaire and GPS. We examined differences in travel duration and distance collected by questionnaire and GPS as well as differences in PB-PAH estimated by these two instruments, thus identifying potential exposure error. We addressed a major gap in the literature, namely, the lack of information on pregnant women’s travel behaviors that influence their exposure to traffic-related air pollutants. Major strengths of the study include the collection of both questionnaire and GPS data, the use of a validated classification model to extract trips from raw GPS data [36], and the quantification of exposure error for an important air pollutant that has been linked to adverse health effects [37,38].

In a previous study [40], we examined the performance of seven portable GPS devices including positional accuracy at stationary locations (e.g. indoor, outdoor) and mobile environments (e.g. walking, traveling by vehicle or bus). The performance of the GPS did not vary substantially inside vs. outside of a purse or bag. On average, higher spatial accuracy was observed for GPS measurements in moving tests than in static tests. Specifically, we found most of the GPS devices performed well for freeway commutes, with 80% of points within 10-m of the route. On surface streets the GPS performance was impacted by surrounding structures in highly urbanized areas. Whereas, we still observed reasonably-well GPS performance for traveling by bus and car in downtown Los Angeles (a challenging environment surrounded by tall buildings), with approximately 90% or more of the points within 20-m of selected surface streets in the area. This is possibly because vehicle or bus routes were relatively away from adjacent buildings (compared to sidewalk), resulting in less blockage or reflection of satellite signals by adjacent buildings. Further, our GPS time-activity classification algorithms considered buffers, speeds, and spatial patterns of GPS points in addition to spatial accuracy and distance to roadways [36]. Thus, we may have captured an even higher percentage of GPS data points for on-road in-vehicle travels.

Limitations in self-reported travel data are well known and primarily attributed to recall bias and rounding inaccuracies in respondents. However, most of the previous travel behavior studies focused on the number of trips and trip distance rather than trip duration. Only a few studies compared and reported discrepancies in travel time between self-reported and corresponding GPS data [27,44-47]. A study in Kentucky, U.S. found that self-reported travel time generally exceeded median GPS-measured values although the difference was much smaller than that for distances [27]. Using part of the data from the 2001 California Statewide Household Travel Survey GPS Study, Wolf et al. [45] observed that on average the self-reported travel time was approximately 38% higher than the measured GPS travel time. A study in Sydney, Australia also reported that people were more likely to overestimate their travel time as measured by GPS [44]. In contrast, a household travel study in Western Cape, South Africa reported under-estimation of trip duration due to rounding of trip departure and arrival times by respondents [47]. The under-estimation was also observed in a Peru study that compared self-reported and GPS-based travel time that agricultural producers needed to get to the nearest population center [46].

In this study we found that subjects overestimated both trip duration and trip distance compared to GPS data, and the over-estimation was more evident in work to home trips (18.5% for trip duration and 13.3% for trip distance) than home to work trips (4.6% for trip duration and 5.6% for trip distance). This is likely because some subjects may have included off-road time (e.g. walking to the vehicle from home or work locations) or short periods of time running errands in the self-reported travel time. For work to home trips, we observed a lower correlation between self-reported and GPS-based trip duration than that of home to work trips (r = 0.64 vs. 0.77). People may at times run errands during their work to home trips rather than going home directly from work, which makes work to home trip less of a routine. Questionnaires should be better formulated in the future to account for other trips that occur during work to home travel. Interestingly, the correlation between self-reported and GPS data was higher for trip distance than trip duration. This is probably because trip distance between a fixed origin and destination (e.g. home and work) was unlikely to change day by day while trip duration was more variable due to the influence of traffic, weather, and other incidental factors that were captured by the GPS but not the self-reported data. We also observed that the subjects tended to report trip duration rounded to the nearest five minutes, quarter, or half hour, which agreed with the previous studies [27].

We found that the study participants on average spent 69 minutes and 93 minutes daily traveling in vehicles based on the GPS and the self-reported data, respectively. The GPS data may have underestimated daily in-vehicle travel time since daily averages were calculated using data on valid GPS days with ≥50% data completeness. By doing so, we likely missed some in-vehicle travel events during the time with no GPS data and thus underestimated total in-vehicle travel time. Klepeis et al. [21] reported an average of 80 minutes per day spent in the in-vehicle microenvironment in California subjects (N = 930) based on diary data from the National Human Activity Pattern Survey (NHAPS). On the other hand, our subjects likely overestimated travel time for non-work related trips. Our interviewers observed that subjects had trouble answering the question on the average daily duration of in-vehicle travel for non-work trips. We asked the subjects to estimate an average of other travel times on a daily basis over a one-week period (Additional file 1). A few subjects were suspected to report total other travel time over a week (e.g. more than 3 hours per day) despite that we asked them a second time to confirm the striking numbers. Since typical non-work related travel do not occur daily, it is challenging for subjects to calculate averages. In future studies, it may be helpful to collect odometer readings of vehicles to estimate total travel distance and non-work travel distance (given work-related travel information from a questionnaire).

The explanatory power was relatively low (about 30% of variance being explained) for models predicting the observed difference in trip duration between self-reported and GPS data. For home to work commutes, trips starting in morning rush hours (6:00 – 8:00 AM) were more likely to have longer GPS travel time compared to self-reported time. A similar result was observed for a different definition of morning rush hours (6:30 – 8:00 AM) for home to work commutes, but rush hours did not enter as a predictor in the models under other definitions (e.g. 6:00 – 9:00 AM and 6:30 – 9:00 AM for home to work commutes). Afternoon rush hours also did not enter as a predictor in the models for work to home commutes (4:00 – 6:00 PM, 4:30 – 6:30 PM, 4:00 – 7:00 PM, and 5:00 – 7:00 PM). Rush hour is an indicator of traffic conditions. However, we could not examine the influence of traffic patterns on the difference of trip duration due to the small sample size (e.g. no trips in certain periods of the day) and unrepresentativeness of the data (pregnant women who likely had different travel patterns than the general population, e.g. more late-afternoon trips than evening trips based on our data). Home to work travel time was more likely to be underestimated in subjects who reported longer trip distance (but not longer trip duration) and overestimated in subjects who used Japanese-made vehicle or had more than two persons in the household. Japanese car owners may be more concerned about fuel efficiency and thus more efficient with errands although we could not verify this from the literature. Subjects with a big family may run errands more frequently during home to work trips than the other subjects, thus, they were prone to overestimate the travel time to potentially account for such incidences. A higher percentage of time on freeways (based on GPS data) was positively associated with the difference for home to work trips, likely because more use of freeways decreased the actual travel time as reflected by the GPS. For work to home travel time, overestimation by subjects was more likely in subjects with longer self-reported travel time, which is expected since subjects who reported longer travel time may have included time running errands (e.g. picking up kids or doing grocery shopping) in work to home travel time. Further, we found self-reported travel time was more likely to underestimate the actual travel time when work to home trips occurred on weekdays than on weekend, likely due to more frequent traffic congestions on weekdays.

We found that at the population level, the estimation of personal exposure to PB-PAH did not differ remarkably (approximately 16% difference) using either self-reported or GPS-based travel time. This estimate improved among women who had both GPS and questionnaire data on work commutes (6.6% difference). However, the exposure estimates differed by as much as three times at the individual level. This inter-individual variation in potential error may produce bias in air pollution epidemiological studies because the relative ranking of individual exposure determines the association between exposure and health outcomes.

We found no substantial differences in daily in-vehicle travel time by pregnancy periods except that the participants tended to spend more time in vehicles in early pregnancy. Longer daily travel time was observed during certain pregnancy periods in women with higher education attainment, higher income, and no children. However, since our sample size was small, this needs to be verified in future studies with more subjects. Other approaches in assessing time in vehicles can be employed as well. For example, in addition to an analyses focusing on instrument comparison and on pregnant women’s travel patterns, we examined the usefulness of a free routing service (e.g. MapQuest: http://www.mapquest.com/) in estimating trip duration based on origin and destination of a trip. More details of the methods, results, and discussion of the results can be found in Additional file 2. Briefly, the models we developed to estimate travel time had strong R^2^ ranging from 0.70 to 0.79, with the most important variable being MapQuest-estimated travel time, which accounted for more than 90% of the variance explained in the models. The free web-based routing service is useful for designing future large epidemiologic studies where GPS is impractical. Using such an approach, obtaining address or location of critical places (e.g. home, work, school, shopping) along with the frequency of each type of travel from a questionnaire may provide more accurate estimates of in-vehicle travel duration and distance than self-reported values.

There were four major limitations in this study. First, we had a greatly reduced sample size in GPS data because of the noncompliance of the subjects and subjects dropping out of the GPS tracking with the advancement in pregnancy. We only obtained 46.7% valid GPS days (≥50% completeness) from the expected days of sampling (549 person-days of valid GPS days from the potential maximum of 56 subjects × 3 trimesters × 7 days = 1176 person-days). The reduced sample size limited the power of stratified analysis. Subjects sometimes forgot to charge the GPS device, or did not turn on the device after battery recharge the next day. This suggests a “missing completely at random” missingness mechanism, which would allow the exclusion of records missing a GPS measurement without bias [48]. We excluded the days with >50% of missing data, but we can’t rule out the possibility that the reason subjects didn’t bring the GPS with them for any particular trip might be related to observed or unobserved factors related to travel times. Based on the meta-analysis of over 15 studies, Krenn et al. 2011 found that longer measurement periods were associated with greater GPS data loss (r = 0.80, p-value < 0.001) and data loss increased substantially after 4 days of sampling [49]. Therefore, future studies should try to minimize the period of sampling if possible; however, this could adversely impact the ability to characterize exposure across different days of week, especially weekday vs. weekend. Data completeness may also be improved by using a GPS instrument with longer battery life or using a GPS cell phone for which subjects may be more likely to charge and keep it with them across locations.

Second, errors may be associated with the GPS trip data extracted from our rule-based time-activity classification model [36]. Errors in time-activity modeling are inevitable although we have carefully validated the model and attempted to minimize the impact of the errors by excluding the trips that lasted less than two minutes. The use of 2003 roadway data might also generate uncertainties in trip classification. The comparison of 2003 and 2005 TeleAtlas roadway data showed only slight differences between the two datasets. Unfortunately, we were not able to obtain roadway data in 2009 when the GPS sampling started; however, we expected no substantial differences between 2003 and 2009 roadway data since our study region (South Los Angeles County and Orange County, California) is a well-developed metropolitan area. Hence, we anticipated minimal influence of using 2003 data on the overall results.

Third, this study was limited by the study design. For instance, our questionnaire asked subjects their travel behaviors in the past 3 months of their pregnancy trimester before the questionnaire date while the GPS tracked their time-location patterns one week following the questionnaire date. The one-week sampling may not be representative of the women’s typical travel patterns in the past 3 months, which would be the exposure period of interest in epidemiologic research of pregnancy-related health outcomes in the mother or child. In addition, women tended to report travel time in a minimum of 5-minute intervals (e.g. 5, 10, and 15 minutes), while the GPS recorded travel time had greater precision. We could not separate these differences between the two instruments. In addition, we did not collect reliable information on vehicle operational factors that may influence in-vehicle exposure (e.g. personal preference on window position, use of air conditioning and recirculation systems), thus we could not incorporate the influence of these factors in the exposure model [18]. If possible, future studies should take into account these influential factors, with a focus on activity that will influence longer-term exposure rather than a snapshot event. Further, since we only focused on work-related commutes in pregnant women, some of the findings may not be generalizable to a larger population including men, children, and non-working pregnant women.

Finally, the majority of our study participants lived in Orange County and Southern Los Angeles County, an area that is not as well served by public transit as other locations. Since most of the study participants were highly dependent on automobiles for their transportation, we were not able to examine the other travel modes (e.g. walking, cycling, bus, or subway) that may also be associated with high levels of exposure to traffic-related air pollutants [50-52].

With the current technology, questionnaire cannot be completely replaced by GPS technologies. Future studies can be improved by increasing the compliance of subjects in GPS data collection (e.g. the use of cell phones to collect data, minimizing subject burden by monitoring only a few days per time, increasing battery life of GPS units), and designing better questionnaire survey [e.g. focusing on routine behaviors, increasing the use of objective measures (e.g. home and work locations), making the questions as easy as possible (e.g. no math calculation to get averages)].

## Conclusion

We found that subjects overestimated both trip duration and trip distance compared to GPS data. Higher correlation was observed for trip distance than trip duration between self-reported and GPS data. Longer daily travel time was observed among participants in early pregnancy, and during certain pregnancy periods in women with higher education attainment, higher income, and no children. Comparing self-reported vs. GPS data, the estimation of personal exposure to PB-PAH did not differ markedly at the population level, but the difference was large at an individual level, which has significant implications in air pollution epidemiological studies. Finally, we found that subject compliance could be a critical issue when relying on GPS alone to collect weekly or longer term time-activity data.

## Consent

Written informed consent was obtained from the participants for the publication of this report.

## Abbreviations

GPS: Portable global positioning system.

## Competing interests

The authors declare that they have no competing interests.

## Authors’ contributions

JW is the PI of this study. JW conceptualized and designed the study, conducted data analyses, and drafted the manuscript. CJ conducted the GPS modeling work and helped with data analysis. GJ recruited human subjects and administered questionnaire interviews and GPS tracking for the majority of the subjects. AD helped to administer questionnaire interviews and GPS tracking for some subjects and helped transfer the paper-based interview data to an electronic database. SB helped with the mixed effects model development and manuscript writing. DB and RD helped conceptualize the study and provided important intellectual content for the manuscript. All authors assisted in the interpretation of results and contributed towards the final version of the manuscript. All authors read and approved the final manuscript.

## Supplementary Material

Additional file 1 Selected questions on socio-demographic status and travel behavior in pregnant women. Click here for file

Additional file 2 Web-based estimate of trip duration and models to estimate GPS-based travel time. Click here for file
