# Supplementary material for: Travel patterns during pregnancy: comparison between Global Positioning System (GPS) tracking and questionnaire data
Source: Environ Health. 2013 Oct 9;12:86. doi: 10.1186/1476-069X-12-86 (PMC3907015; doi:10.1186/1476-069X-12-86)
Supplement: Additional file 1 — Selected questions on socio-demographic status and travel behavior in pregnant women. [file 1476-069X-12-86-S1.pdf]

Selected questions on socio-demographic status and travel behavior in pregnant women

BASELINE INFORMATION

1. **When is your last menstrual period?**
   Year__________ Month_________Day__________

2. **Based on the doctor's diagnosis, how far along have you been pregnant?**
   _________weeks

3. **Did you ever have any other babies who were born alive?**
   - ☐ Yes **(If yes, how many? ____________)**
   - ☐ No (If No, skip to question 17)
   - ☐ Refused
   - ☐ Don't know

4. **How tall are you without shoes or what is your height on your driver's license?**
   - ☐ ____ feet ____ inches
   - ☐ Refused
   - ☐ Don't know

5. **How much did you weigh before you became pregnant?**
   - ☐ ______ pounds
   - ☐ Refused
   - ☐ Don't know

6. **What is your ethnic or racial background? (Mark al that apply)**
   - ☐ White
   - ☐ Black (African-American)
   - ☐ Asian
   - ☐ Native Hawaiian or other Pacific Islander
   - ☐ North American Indian (Native American)
   - ☐ Other (Explain: _________________)

7. **Are you of Hispanic or Spanish or Latino descent?**
   - ☐ Yes
   - ☐ No

8. **Do you speak a language besides English?**
   - ☐ No
   - ☐ Yes.
     ⇓
     Which language?
     - ☐ Spanish
     - ☐ Other (specify: ______________________)

9. **What is the highest degree you have earned?**
   - ☐ Did not finish grade school (1-8)
   - ☐ Grade school (1-8)

- ☐ High school diploma
- ☐ Technical or trade school diploma
- ☐ College diploma
- ☐ Graduate school diploma
- ☐ Other (specify) ___________
- ☐ Refused
- ☐ Don’t know

10. **What is your marital status?**
    - ☐ Single (never married)
    - ☐ Living with someone, but not legally married
    - ☐ Married
    - ☐ Separated
    - ☐ Divorced
    - ☐ Widowed
    - ☐ Other (specify): ___________________
    - ☐ Refused
    - ☐ Don’t know

11. **I don’t need to know exactly, but what was your household’s approximate income from all sources in 2008, before taxes? Was it…**
    - ☐ Less than $10,000
    - ☐ $10,000 to less than $20,000
    - ☐ $20,000 to less than $30,000
    - ☐ $30,000 to less than $40,000
    - ☐ $40,000 to less than $50,000
    - ☐ $50,000 to less than $75,000
    - ☐ $75,000 or more
    - ☐ Refused
    - ☐ Don’t know

12. **How many people living in your household were supported by this income (including yourself)?**
    - ☐ __________ (total number of people)
    - ☐ Refused
    - ☐ Don’t know

13. **How many of these people were children under the age of 18?**
    - ☐ __________ (total number of children)
    - ☐ Refused
    - ☐ Don’t know

HOME LOCATION(S) AND COMMUTING

Did you move to a different home in the past THREE months?

☐ No → Please fill in **Column 1** of the table below
☐ Yes, I moved once → Please fill in **Columns 1 and 2** of the table below

Question	Column 1<br>**FIRST HOME you lived**	Column 2<br>**SECOND HOME you lived**
14. **Duration of time**	Date moved in? _________ month/day/year<br>☐ Don't know	Date moved in?_________ month/day/year<br>☐ Don't know
15. **Street Address**		
16. **City**		
17. **State, Zip Code**		
18. **Did you work outside this home?**	☐ Yes<br>☐ Don't know<br>☐ No → If no, go to question **9**	☐ Yes<br>☐ Don't know<br>☐ No → If no, go to question **9**
19. **How did you usually get to work from this home? (Interviewer: check all that apply and if >1 take notes on how transport was combined)**	☐ Automobile ☐ Walk<br>☐ Metro rail or train ☐ Bus<br>☐ Bicycle ☐ Motorcycle<br>☐ Other: _________________<br>☐ Don't know	☐ Automobile ☐ Walk<br>☐ Metro rail or train ☐ Bus<br>☐ Bicycle ☐ Motorcycle<br>☐ Other: _________________<br>☐ Don't know
20. **Around how far did you typically have to travel from this home to work? Include the miles from any routine stops you made on the way to work: for example, day care or school. If you worked in different locations, please tell us the total number of miles you traveled on a typical day.**	________ miles (going to work)<br>________ miles (returning from work)<br>☐ Don't know<br>***Interviewer*: Please determine the typical travel route from this home to work and back using main highways and freeways (skip small streets) or metro rail or train route. Obtain details for each place of work, including usual schedule, location.**	________ miles (going to work)<br>________ miles (returning from work)<br>☐ Don't know<br>***Interviewer*: Please determine the typical travel route from this home to work and back using main highways and freeways (skip small streets) or metro rail or train route. Obtain details for each place of work, including usual schedule, location.**

Question	Column 1	Column 2
	**FIRST HOME you lived**	**SECOND HOME you lived**
21. **How much time did you spend commuting to work from this home during a typical workday? Include routine stops you made on the way to work; for example, day care or school. At what times of day did you usually go to work and return from work. If you worked in different locations, please tell us the amount of time it took you to get to work on a typical day, and at what time of day. Please give a one-way estimate, not round-trip.**	**Workplace 1**<br>________ minutes (going to work)<br>Departure time: ______hh:mm __AM __ PM<br>________ minutes (returning from work)<br>Departure time: ______hh:mm __AM __ PM<br>☐ Don't know<br>**Workplace 2**<br>________ minutes (going to work)<br>Departure time: ______hh:mm __AM __ PM<br>________ minutes (returning from work)<br>Departure time: ______hh:mm __AM __ PM<br>☐ Don't know	**Workplace 1**<br>________ minutes (going to work)<br>Departure time: ______hh:mm __AM __ PM<br>________ minutes (returning from work)<br>Departure time: ______hh:mm __AM __ PM<br>☐ Don't know<br>**Workplace 2**<br>________ minutes (going to work)<br>Departure time: ______hh:mm __AM __ PM<br>________ minutes (returning from work)<br>Departure time: ______hh:mm __AM __ PM<br>☐ Don't know
22. **On average, how much time did you spend in vehicles (cars or buses) other than trips from home to work and back while you were pregnant?**	________ minutes<br>________ % of that time on freeways<br>☐ Don't know<br>***Interviewer*: Please describe the typical travel route from each home (first through third) to specific common locations using main highways and freeways (skip small streets) or metro rail or train route. Obtain details for each place including mode of travel, time of day, frequency per day, week.**	________ minutes<br>________ % of that time on freeways<br>☐ Don't know<br>***Interviewer*: Please describe the typical travel route from each home (first through third) to specific common locations using main highways and freeways (skip small streets) or metro rail or train route. Obtain details for each place including mode of travel, time of day, frequency per day, week.**

PERSONAL VEHICLE

23. Did you have a car available for your transportation while you were pregnant? This includes the main car used to transport you to work or other common locations even if you did not drive it yourself (e.g., your husband's car).

☐ **Yes**

☐ **No** → If no, go to question 12 Interviewer: please clarify the extent to which the subject did not ride in a car for transportation (e.g., only used public transport, occasionally rode with a friend, etc.).

☐ Don't know

What was the model and year of this car?

Make: __________________ (e.g., Ford) ☐ Don't know

Model: __________________ (e.g., Explorer) ☐ Don't know

Enter year: ________________ or if you don't know the year

Enter decade: ______________ (e.g., 1970's, 1980's) ☐ Don't know

Approximate Odometer reading _______________ miles ☐ Don't know

<u>OCCUPATIONAL EXPOSURE DURING PREGNANCY:</u> one set each for Workplace 1-2 (pages 5-7)

Workplace 1:

24. What is your job title or job description? **(E.g., accountant, secretary, teacher, etc.)**

Note: If you have more than one job, please provide the title or description for your *main job*, i.e., the job where you work the longest and for the most hours.

Job title/description: ______________________________

☐ Don’t know

25. Where is this job located?

List the address of the building (you do not need to list the name of the company – only the number and street name for the building):

Address: ____

City: ____

State:_________ Zip Code: ____________________

Or list the nearest two cross streets and city, if you cannot remember the address:

___________________ and _______________________

City: __________________________________

☐ Don’t know

26. How long have you worked at this job?

☐ __________ years

☐ __________ months

☐ Don’t know

27. When do you plan to stop working at this job for maternal leave?

☐ I will work until I deliver

☐ I will stop working 1 month before I deliver.

☐ I will stop working 2 months before I deliver.

☐ I will stop working 3 months or more before I deliver…

☐ Don’t know

☐ Other (interviewer fill in): _____________________________________

28. What is your typical working hours at this job?

Starting time: _____:_____ AM / PM *(please circle AM or PM)*

Ending time: _____:_____ AM / PM *(please circle AM or PM)*

29. What are your typical work days of the week at this job? Please check ALL that apply.

Monday	Tuesday	Wednesday	Thursday	Friday	Saturday	Sunday

Interviewer: Note different start times by day of week if more than one hour different from typical hours.
