# Supplementary material for: Travel patterns during pregnancy: comparison between Global Positioning System (GPS) tracking and questionnaire data
Source: Environ Health. 2013 Oct 9;12:86. doi: 10.1186/1476-069X-12-86 (PMC3907015; doi:10.1186/1476-069X-12-86)
Supplement: Additional file 2 — Web-based estimate of trip duration and models to estimate GPS-based travel time. [file 1476-069X-12-86-S2.docx]

***Web-based estimate of trip duration***

**Methods**

We used the free MapQuest routing service (<http://www.mapquest.com/>) to estimate trip duration based on origin and destination coordinates for the GPS-identified trips lasting two minutes or longer. We used the same automation approach as that in McConnell et al. (2010) to send requests to and extract travel time responses from the MapQuest service for the large number of trips. The estimated time did not take into account real-time traffic conditions including traffic congestion. The routing service can use both address and coordinate information as input parameters. We extracted the first and the last point of each GPS-identified trip as the origin and destination locations and used them as inputs to obtain web-based estimate of trip duration. The web routing service calculated optimum travel routes of the shortest time by measuring distances and assigning appropriate travel speeds on different roadways. We used the MapQuest service in this paper because we have used it before and it provided an application programming interface (API) that can be adapted easily by the users to submit and extract queries automatically. We previously found that trip duration estimates from the MapQuest and the Google Earth were highly correlated with correlation >0.95 (data not shown).

We developed linear regression models and linear mixed effect models to predict GPS-based trip duration and total daily travel time using web-based trip duration estimates based on the origin and destination coordinates of the trips (the individual trip data were summed up on a daily basis for the daily travel time models). The other variables we examined in the models included questionnaire-based travel information (e.g. self-reported distance and duration of work-related trips, self-reported daily travel time of non-work related trips and the percentage of travel time on freeways), socio-demographic variables (i.e. age, working status, education, income, marital status, and parity), and other factors that may influence travel time (i.e. season, day of week, rush hour, and the percent of travel time on freeways based on GPS data). The following parameters were treated as binary variables in the models: season (cool or warm), day of week (weekday or weekend), trip staring in rush hour (yes or no), age (<30 or ≥30), household income (<$50 k or ≥$50 k), marital status (yes or no), and parity (0 or ≥1). We classified May to October as warm season and November to April as cool season. We define rush hour as 6 AM – 8 AM and 4 PM – 6 PM. The following parameters were treated as continuous variables in the models: MapQuest-estimated and self-reported trip distance and duration, and self-reported and GPS-derived percent of travel time on freeways.

**Results**

Table A1 shows the modeling results for the prediction of GPS-based in-vehicle travel time at the trip level. The linear regression and the mixed effect model produced similar results; the linear model had a cross validation R^2^ of 0.71 and a RMSE of 8.87. On average, GPS travel time was about 32% longer than the MapQuest-estimated travel time; trip started in rush hour lasted slightly longer (2.7 minutes) than the other trips; each one minute of self-reported home to work trip duration increased GPS travel time by 0.015 minute; and the percent of time on freeways increased the GPS travel time by up to 5 minutes. MapQuest-estimated travel time interacted with the percent of travel time on freeways. For individual trips where there was 0% of time on freeways, GPS travel time was about 32% longer than the MapQuest estimate, but for individual trips where there was 100% of time on freeway GPS travel time was about 15% shorter, suggesting that MapQuest estimates were better for trips on freeways than those on surface streets. No demographic and SES variables were entered into the model. All of the likelihood ratio tests had p-values <0.0001, indicating that a random effect models fit the data better than the linear models.

Table A2 shows the models for predicting daily in-vehicle travel time. The models were built using GPS travel time averaged by person weeks with at least 3 valid GPS days per week. MapQuest-estimated travel time and percent of trips starting in rush hour explained 72% of the variance in daily travel time in the linear regression model. The R^2^ only decreased slightly for the cross-validation results. The mixed effect models also showed results that were not markedly different than the linear models although mixed effect models showed better model fit than the linear models (likelihood ratio tests had p-values <0.0001).

Table A1. Linear and mixed effect models to predict GPS-based travel time for all trips > 2 minutes using web-based travel time estimates (N=1728 trips for all the subjects).

|  | Linear regression | | | | | Mixed effect model (fixed effects) | | |
| --- | --- | --- | --- | --- | --- | --- | --- | --- |
|  | Beta | SE*^a^* | p-value | Model R^2^  (CV R^2^) *^b^* | RMSE*^c^* | Beta | SE*^a^* | p-value |
| Intercept | 1.12 | 0.44 | 0.02 | 0.71 (0.70) | 8.87 | 0.89 | 0.51 | 0.08 |
| MapQuest-estimated trip duration using GPS-based trip origin and destination (minutes) | 1.23 | 0.039 | <.0001 |  |  | 1.32 | 0.052 | <.0001 |
| Trip started in rush hour (1/0)*^d^* | 3.23 | 0.48 | <.0001 |  |  | 2.71 | 0.46 | <.0001 |
| Percent of time on freeways | 6.57 | 1.23 | <.0001 |  |  | 5.42 | 1.31 | <.0001 |
| Self-reported home to work trip duration (minutes) | 0.028 | 0.0076 | 0.0002 |  |  | 0.015 | 0.0095 | 0.11 |
| MapQuest trip duration: percent of time on freeways | -0.44 | 0.065 | <.0001 |  |  | -0.48 | 0.073 | <.0001 |

*^a^*Standard error; *^b^*Leave-one-out cross validation R^2^; *^c^*Square root of the mean of the squared errors; *^d^*Rush hour was defined as trip starting time of 6 AM – 8 AM and 4 PM – 6 PM.

Table A2. Linear and mixed effect models to predict GPS-based daily in-vehicle travel time averaged over a minimum of three valid GPS days per sampling week (N=83 person weeks).

|  | Linear regression | | | |  | Mixed effect model (fixed effects) | | |
| --- | --- | --- | --- | --- | --- | --- | --- | --- |
|  | Beta | SE*^a^* | p-value | Model R^2^  (CV R^2^) *^b^* | RMSE*^c^* | Beta | SE | p-value |
| Intercept | 8.93 | 5.54 | 0.11 | 0.72 (0.68) | 19.32 | 13.62 | 5.98 | 0.0272 |
| MapQuest-estimated travel time*^d^* | 1.15 | 0.083 | <.0001 |  |  | 1.12 | 0.09 | 0.0000 |
| Percent of trips starting during rush hour*^e^* | 31.10 | 13.95 | 0.0286 |  |  | 18.60 | 11.08 | 0.1029 |

*^a^*Standard error; *^b^*Leave-one-out cross validation R^2^; *^c^*Square root of the mean of the squared errors; *^d^*Sum of MapQuest-estimated trip durations based on the origins and destinations of GPS-derived trips; *^e^* Rush hour was defined as trip starting time in 6 AM – 8 AM and 4 PM – 6 PM.
